# Design and synthesis of hierarchical NiO/Ni_3_V_2_O_8_ nanoplatelet arrays with enhanced lithium storage properties†

**DOI:** 10.1039/c9ra08252b

**Published:** 2019-12-02

**Authors:** Yang Li, Feng Duan, Shuai Yang, Qihuang Deng, Songli Liu, Cheng Peng

**Affiliations:** College of Materials Science and Engineering, Yangtze Normal University Chongqing 408100 People's Republic of China yangli_yznu@163.com +86-23-72790029 +86-23-72790029

## Abstract

Hierarchical NiO/Ni_3_V_2_O_8_ nanoplatelet arrays (NPAs) grown on Ti foil were prepared as free-standing anodes for Li-ion batteries (LIBs) *via* a simple one-step hydrothermal approach followed by thermal treatment to enhance Li storage performance. Compared to the bare NiO, the fabricated NiO/Ni_3_V_2_O_8_ NPAs exhibited significantly enhanced electrochemical performances with superior discharge capacity (1169.3 mA h g^−1^ at 200 mA g^−1^), excellent cycling stability (570.1 mA h g^−1^ after 600 cycles at current density of 1000 mA g^−1^) and remarkable rate capability (427.5 mA h g^−1^ even at rate of 8000 mA g^−1^). The excellent electrochemical performances of the NiO/Ni_3_V_2_O_8_ NPAs were mainly attributed to their unique composition and hierarchical structural features, which not only could offer fast Li^+^ diffusion, high surface area and good electrolyte penetration, but also could withstand the volume change. The *ex situ* XRD analysis revealed that the charge/discharge mechanism of the NiO/Ni_3_V_2_O_8_ NPAs included conversion and intercalation reaction. Such NiO/Ni_3_V_2_O_8_ NPAs manifest great potential as anode materials for LIBs with the advantages of a facile, low-cost approach and outstanding electrochemical performances.

## Abstract 2

Hierarchical NiO/Ni_3_V_2_O_8_ nanoplatelet arrays (NPAs) grown on Ti foil were prepared as free-standing anodes for Li-ion batteries (LIBs) *via* a simple one-step hydrothermal approach followed by thermal treatment to enhance Li storage performance.

## Graphical abstract

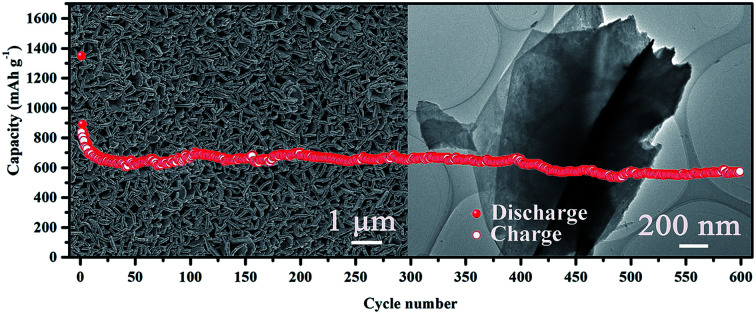

## 1 Introduction

Li-ion batteries (LIBs) have attracted much interest as electrochemical energy storage devices due to their outstanding performances in terms of high energy density, high voltage, long lifespan and environmental benignity.^1–3^ However, conventional graphite anodes have a low theoretical capacity (372 mA h g^−1^), which hardly meets the growing energy demand for various consumer electronic devices. In the past few decades, extensive research has been devoted to develop alternative electrode materials aiming for higher energy/power density, longer cycle life, increased safety and lower cost. Nowadays, transition metal oxides (TMOs) exhibit great specific capacities, high volumetric energy densities and intrinsically enhanced safety, making them the supposed alternative anode materials for LIBs.^4–8^

Nickel oxide (NiO) was one of the distinguished candidates, which was not only easy to synthesize, but also had high theoretical capacity (718 mA h g^−1^) with great chemical/thermal stability.^9,10^ However, its low electronic/ionic conductivity and structure destruction in cycling hindered the application of NiO anodes in LIBs. One attractive strategy toward assuaging these problems is to build various nanostructured electrode materials, such as nanoparticles,^11,12^ nanospheres,^13^ nanorodes^14^ and nanosheets.^15,16^ Nanostructured electrodes materials can alleviate the stress-induced structural variation derived from repeated lithiation/delithiation process, and provide short diffusion path for electron/ion transfer compared with their bulk counterparts.^17–20^ Remarkably, the electrode materials aligned directly on current collectors with hierarchical nanostructure exhibit outstanding electrochemical activities, due to the potential for providing a larger surface area to improve the interfacial kinetics, more space to buffer the volume variation and easier pathways for electrolyte penetration.^21–23^

Another effective approach is to rely largely on designing different ternary transition metal oxides by introducing various metal species into target products, which can synergistically enhance electrochemical properties owning to their enhanced electrochemical activities, electrical conductivity and mechanical stability.^24,25^ The nickel-based ternary transition metal oxides, such as NiCo_2_O_4_,^26^ NiMn_2_O_4_ (ref. 27) and NiMoO_4_ (ref. 28) have been examined as potential anode materials for LIBs. Among them, Ni_3_V_2_O_8_ has a wide range of energy storage applications.^29,30^ For example, ordered mesoporous carbon supported Ni_3_V_2_O_8_ composites,^31^ Ni_3_V_2_O_8_ amorphous wire encapsulated in crystalline tube nanostructure,^32^ Ni_3_V_2_O_8_/carbon cloth hierarchical structures^33^ showed great storage capacities, holding great promise in LIBs.

Recently, various hybridizing metal oxides with an integrate nanostructure have attracted much attention in exploring alternative anode materials for LIBs as they can take advantage of interaction of different components for enhanced electrochemical performances.^34–39^ For instance, Lou *et al.*^40^ successfully synthesized a novel Co_3_O_4_@Co_3_V_2_O_8_ hollow structure with a metal organic framework, which exhibited superior electrochemical activity. Bases on the appealing concept, many NiO-based nanocomposites, such as NiO–Co_3_O_4_ nanoplate,^41^ porous NiO–ZnO hybrid nanofibers^42^ and hierarchical porous NiO–NiMoO_4_ heterostructure^43^ have been fabricated and tested as high-performance anode materials for LIBs. Only very recently, P. Vishnukumar *et al.*^44^ successfully synthesized NiO/Ni_3_V_2_O_8_ nanocomposite by a solvothermal method, which exhibited outstanding super-capacitive activity. However, its application for LIBs is rarely reported until now. Furthermore, there is no research on the direct growth of NiO/Ni_3_V_2_O_8_ hierarchical nanostructures on conductive collectors for superior lithium storage properties.

In this study, we described a facile one-step hydrothermal approach to fabricate a hierarchical NiO/Ni_3_V_2_O_8_ nanoplatelet arrays (NPAs) directly grown on Ti foil, followed by thermal treatment. In this architecture, the interpenetrated NiO/Ni_3_V_2_O_8_ nanoplatelets assembled into a framework, in which the NiO and Ni_3_V_2_O_8_ were homogeneously dispersed at the nanoscale. The newly synthesized NiO/Ni_3_V_2_O_8_ NPAs benefited from novel *in situ* electrochemical reconstruction exhibited high specific capacity, excellent cycling stability and great storage capability. The mechanism of lithiation/delithiation of new NiO/Ni_3_V_2_O_8_ NPAs was also explored *via ex situ* XRD measurements, making contributions to the application of transition metal oxides for energy storage.

## 2 Experimental sections

### 2.1 Synthesis of NiO/Ni_3_V_2_O_8_ NPAs

**Fig. 1:**
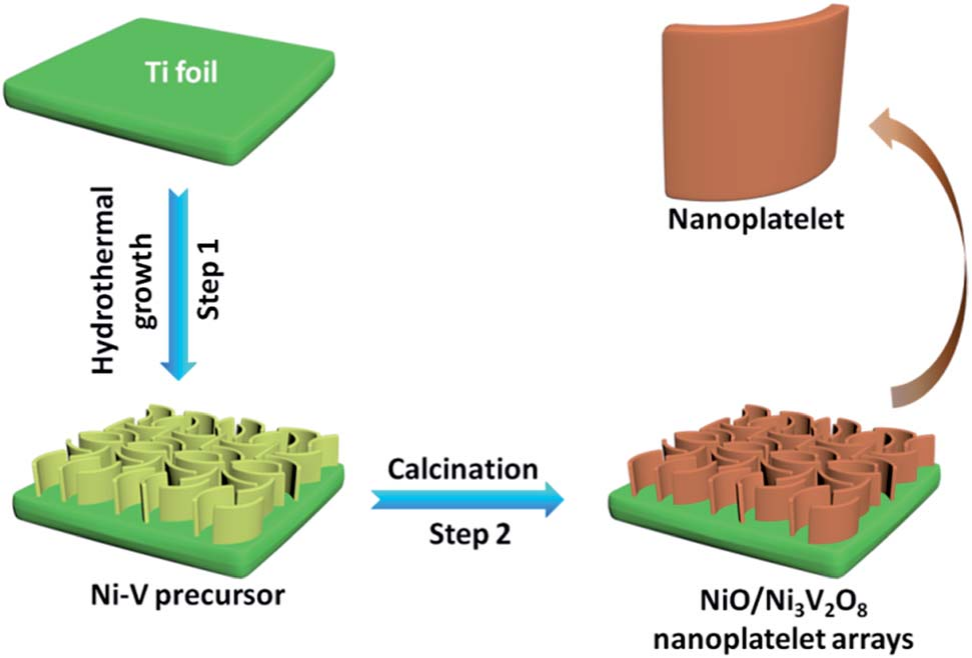
Illustration of NiO/Ni_3_V_2_O_8_ NPAs formation process.

All the reagents, HCl (3 M), ethanol, acetone and deionized water, used in the study were of analytical grade to eliminate impurities on the surface of Ti foil. In a typical synthesis, 1 mmol Na_3_VO_4_ and 1 mmol NiCl_2_·6H_2_O were dissolved into 80 mL deionized water with vigorous magnetic stirring. Subsequently, 7 mmol NiCl_2_·6H_2_O and 50 mmol CO(NH_2_)_2_ were dissolved in another fresh 80 mL distilled water followed by adding above solution under stirring. The mixed solution was continuous stirring for 15 min and then transferred into a 200 mL Teflon-lined autoclave with inserting a piece of clean Ti foil and maintained at 160 °C for 8 h. The resultant substrate was fetched out and washed with distilled water several times, then dried in oven at 60 °C and annealed at 400 °C in the air for 3 h to obtain the NiO/Ni_3_V_2_O_8_ NPAs. As a comparison, the bare NiO samples were also prepared under the same condition without the addition of Na_3_VO_4_ in precursor solution. The integrated synthesis process for the NiO/Ni_3_V_2_O_8_ NPAs was depicted in Fig. 1.

### 2.2 Materials characterization

The crystal phase of the product was characterized by X-ray diffraction patterns (XRD, Rigaku D/MAX 2400). The morphology and microstructure of the specimen was characterized by Scanning Electron Microscope (SEM, JEOL, JSM-6701F) and Transmission Electron Microscope (TEM, JEOL, JEM-2010) with Energy Dispersive Spectrometer (EDS). The composition of the sample was analyzed by Inductively Coupled Plasma (ICP-AES, IRIS Intrepid II XSP).

### 2.3 Electrochemical measurements

The electrochemical performance was measured by CR2032 coin cells assembled with the NiO/Ni_3_V_2_O_8_ as working electrode, Li foil, Celgard 2300 membrane and electrolyte in a mixture of 1 M LiPF_6_ in ethylene carbonate (EC) and dimethyl carbonate (DMC) (1 : 1, by volume). The mass loading of the active materials was around 1–1.3 mg cm^−2^. Cyclic voltammetry (CV) was carried out on an electrochemical workstation (CHI660E). The galvanostatic charging–discharging test was performed on the Li-ion battery cycler (LAND CT2001A) at different current rates. The electrochemical impedance spectroscopy (EIS) was measured on an electrochemical workstation (CHI660E).

## 3 Results and discussion

### 3.1 Synthesis and characterization

**Fig. 2:**
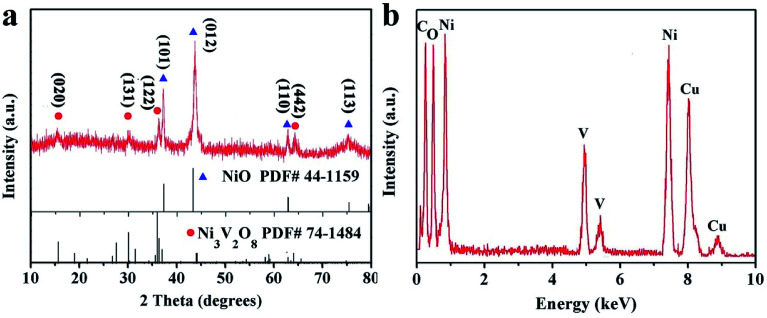
(a) XRD and (b) EDS pattern of NiO/Ni_3_V_2_O_8_ NPAs.

Fig. 2a showed the XRD pattern of NiO/Ni_3_V_2_O_8_ NPAs. The diffraction peaks were located at 37.2, 43.2, 62.8 and 74.4 degree ascribing to the (101), (012), (110) and (113) faces of NiO (JCPDF card no. 44-1159), whereas the diffraction peaks were located at 15.5, 29.9, 35.9 and 64.0 degree originating from the phase of Ni_3_V_2_O_8_ (JCPDF card no. 74-1484). There were no other detectable phases. Furthermore, the results of EDS (Fig. 2b) confirmed that the specimen was only constituted with NiO and Ni_3_V_2_O_8_. The Ni/V atomic ratio determined by ICP technique of two specimens was about 7.58 : 1 (Table S1†), corresponding to 30.8% (mass percentage) of Ni_3_V_2_O_8_ in NiO/Ni_3_V_2_O_8_ NPAs.

**Fig. 3:**
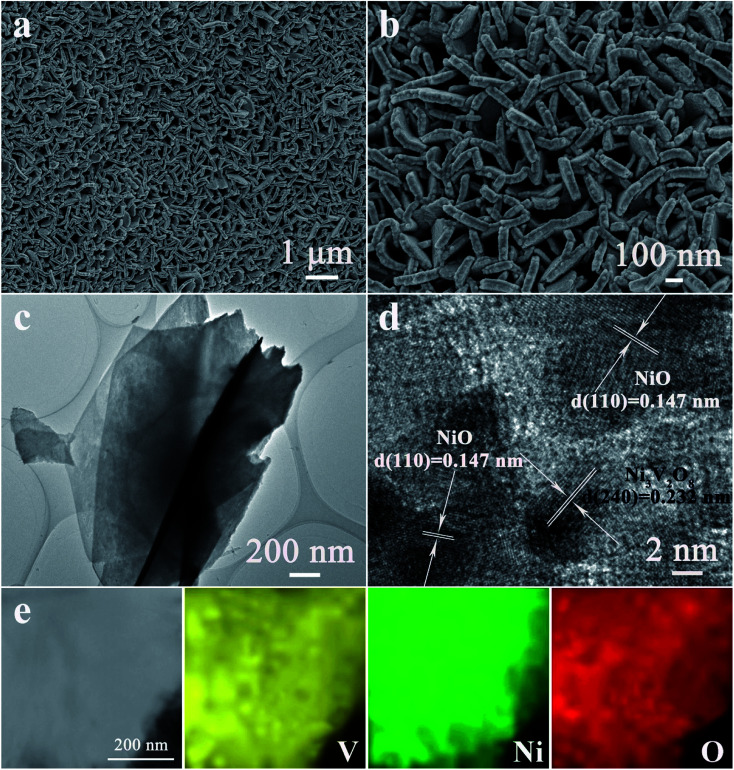
(a and b) SEM and (c) TEM images of NiO/Ni_3_V_2_O_8_ NPAs. (d) High resolution TEM image of NiO/Ni_3_V_2_O_8_ NPAs. (e) The scan TEM image and the corresponding EDS elemental mapping images of Ni, V, and O.

The morphology and microstructure of the as-fabricated NiO/Ni_3_V_2_O_8_ NPAs on Ti foil were characterized by SEM and TEM. Fig. 3a and b showed that the NiO/Ni_3_V_2_O_8_ NPAs had an open and highly porous framework structure assembled by interpenetrated nanoplatelets, homogeneously anchored on the surface of Ti foil. In contrast, the NiO samples displayed a bulk structure with size of around several microns as shown in Fig. S1.† The TEM image (Fig. 3c) further verified the nanoplatelet structure of NiO/Ni_3_V_2_O_8_ NPAs, which was consistent with SEM result obtained from Fig. 3b. The thickness of a single NiO/Ni_3_V_2_O_8_ nanoplatelet was estimated to be about 60–80 nm. The high-resolution TEM image of NiO/Ni_3_V_2_O_8_ NPAs (Fig. 3d) showed that the lattice spacings were 0.147 and 0.232 nm for the (110) crystal planes of NiO, and (240) crystal planes of Ni_3_V_2_O_8_, respectively. The corresponding selected-area electron diffraction (SEAD) pattern showed that the NiO/Ni_3_V_2_O_8_ NPAs were well crystallized and polycrystalline (Fig. S2†). The scan TEM image and the EDS mappings of NiO/Ni_3_V_2_O_8_ NPAs (Fig. 3e) revealed the uniform distribution of Ni, V and O elements. This result confirmed that the NiO and Ni_3_V_2_O_8_ subunits were homogeneously dispersed in the NiO/Ni_3_V_2_O_8_ NPAs and contacted with each other intimately.

### 3.2 Electrochemical performances of the electrodes

**Fig. 4:**
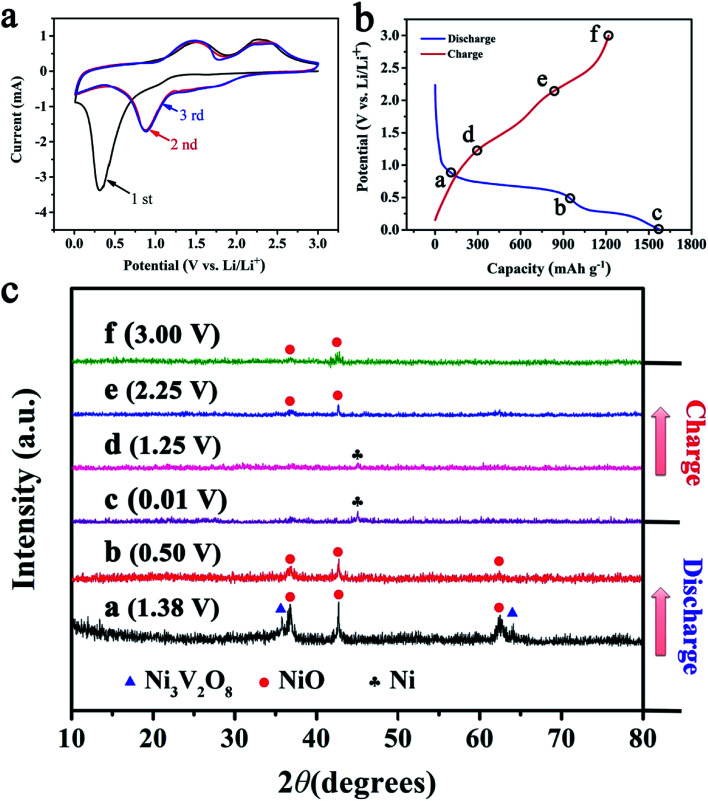
(a) The CV curves of NiO/Ni_3_V_2_O_8_ nanohybrides for the first three cycles. (b and c) The *ex situ* XRD patterns of NiO/Ni_3_V_2_O_8_ nanohybrides under different discharge and charge states.

The electrochemical performance of NiO/Ni_3_V_2_O_8_ NPAs was tested as an anode for LIBs. Fig. 4a showed CV cures of NiO/Ni_3_V_2_O_8_ NPAs ranged from 0.01 to 3.0 V (*vs.* Li/Li^+^) with 0.1 mV s^−1^ scan rate. During the first discharge cycle, a broad reduction peak was observed at around 1.38 V, which might result from the reduction of Ni_3_V_2_O_8_ into NiO companied with the formation of Li_*x*_V_2_O_5_.^45^ The broad irreversible cathodic peak was located at 0.75–0.01 V corresponding to the reduction of NiO to Ni, the insertion of Li^+^ into Li_*x*_V_2_O_5_ and the formation of solid electrolyte interface (SEI).^31^ The reduction peaks shifted to 1.89 V, 1.03 V and 0.72 V in the subsequent cycles, which could be attributed to the possible activation process as described by Sambandam *et al.*,^46^ including the phase transformation, structural reorganization and a polarization change in the sample material. During the first charging cycle, the oxidation peak was observed at around 1.25 V, corresponded to the oxidation of Ni into NiO, and two weak reduction peaks was located at 2.0–3.0 V attributing to the extraction of Li^+^ from Li_*x*_V_2_O_5_. In addition, the overlapped CV curves from the second cycle onward indicated a good stability and cyclability for the insertion and extraction of Li^+^.

In order to investigate the mechanism of lithiation/delithiation of NiO/Ni_3_V_2_O_8_ NPAs, a series of partially lithiated NiO/Ni_3_V_2_O_8_ NPAs with various charge and discharge states (denoted with letters “a” to “f” in Fig. 4b and c) during the first cycle were examined by the *ex situ* X-ray (Fig. 4c). Fig. 4c-a demonstrated that the Ni_3_V_2_O_8_ phase gradually turned to the NiO phase (JCPDF card no. 44-1159) and amorphous Li_*x*_V_2_O_5_ with discharging from 3.00 V to 1.38 V. Then, the intensity of NiO at the peak reduced sharply, and the diffraction peaks of Ni_3_V_2_O_8_ disappeared when discharging to 0.50 V (Fig. 4c-b), implying the conversion reaction of NiO to Ni accompanied with the reduction of all the Ni_3_V_2_O_8_ into NiO and Li_*x*_V_2_O_5_. After discharging to 0.01 V (Fig. 4c-c), the peak of NiO disappeared and a new phase of Ni (JCPDF card no. 03-1051) appeared, which corresponded to a conversion reaction of NiO to Ni and the insertion of Li^+^ into Li_*x*_V_2_O_5_. Finally, the peak of Ni gradually disappeared and the NiO phase reappeared with increased charging degree (Fig. 4c-d and f), which could attribute to the oxidation of Ni into NiO and the extraction of Li^+^ from Li_*x*_V_2_O_5_.

As discussed above, the electrochemical mechanism of NiO/Ni_3_V_2_O_8_ NPAs based on conversion and intercalation reaction could be represented as follows:

1 NiO + Ni_3_V_2_O_8_ + *x*Li^+^ + *x*e^−^ → 4NiO + Li_*x*_V_2_O_5_

2 NiO + 2Li^+^+ 2e^−^ ↔ NiO + Li_2_O

3 Li_*x*_V_2_O_5_ + *y*Li^+^ + *y*e^−^ ↔ Li_*x*+*y*_V_2_O_5_

**Fig. 5:**
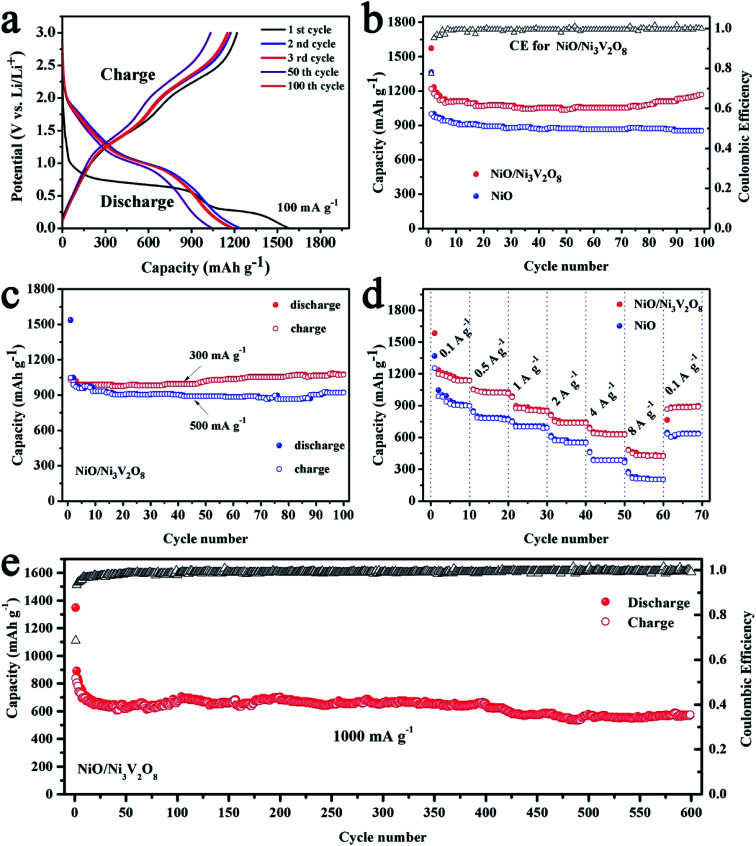
(a) The charge–discharge profiles of NiO/Ni_3_V_2_O_8_ NPAs. (b) Comparison of cycling performance of NiO/Ni_3_V_2_O_8_ NPAs and NiO samples. (c) The cycling performance of NiO/Ni_3_V_2_O_8_ NPAs at 300 and 500 mA g^−1^. (d) Rate capability testing at various current densities. (e) Long cycling performance of the NiO/Ni_3_V_2_O_8_ NPAs at a large specific current of 1000 mA g^−1^.

The discharge–charge cycling test were carried out at 100 mA g^−1^ with the voltage window of 0.01–3 V to assess the cycle performance of the NiO/Ni_3_V_2_O_8_ NPAs (Fig. 5a). Results showed that there were two discharge potential plateaus located at 1.75 V and 0.25 V during the first discharge process. The initial discharge and charge capacities were 1572.4 and 1366.2 mA h g^−1^, respectively, corresponding to an irreversible capacity of 13.1%. The large capacities loss was common for metal-oxide based anodes due to the formation of SEI film caused by electrolyte degradation.^47,48^ Meanwhile, the capacity of the NiO/Ni_3_V_2_O_8_ NPAs in the 100th cycle was similar to the 3rd cycle and higher than that of 50th cycle, representing the good stability of the NiO/Ni_3_V_2_O_8_ NPAs. It is interesting that the specific capacities of the NiO/Ni_3_V_2_O_8_ NPAs decreased slowly in initial 50 cycles, and then increased gradually, finally reached up to 1169.3 mA h g^−1^ after 100 cycles as demonstrated in the cycling performance for NiO/Ni_3_V_2_O_8_ NPAs at 100 mA g^−1^ (Fig. 5b). The increasing capacity might originate from gradual participation in electrochemical reaction of active NiO/Ni_3_V_2_O_8_ NPAs along with cycling number.^49^ The coulombic efficiency (CE) of initial cycle for NiO/Ni_3_V_2_O_8_ NPAs was 86.9%. After that, the values of CE increased significantly, and then kept stable in subsequent cycles, suggesting good capacity recovery ability. Fig. 5c showed that the initial discharge capacities were 1538.3 and 1535.1 mA h g^−1^ at current of 300 and 500 mA g^−1^, and retained at 1076.5 and 922.1 mA h g^−1^, after 100 cycles, respectively. As a comparison, the bare NiO samples only delivered a low capacity of 852.8 mA h g^−1^ after 100 cycles at the current density of 100 mA g^−1^. In summary, the as-prepared NiO/Ni_3_V_2_O_8_ NPAs showed distinct enhancement over the reported NiO-based and Ni_3_V_2_O_8_-based anodes as summarized in Table S2.†^32,37,41,42,50,51^

Results from the rate performance of the NiO/Ni_3_V_2_O_8_ NPAs (Fig. 5d) showed that the discharge capacities of NiO/Ni_3_V_2_O_8_ NPAs were 1138.4, 1026.9, 857.5, 744.4 and 634.2 mA h g^−1^ at the current densities of 100, 500, 1000, 2000 and 4000 mA g^−1^, respectively. Even at a high rate of 8000 mA g^−1^, a large capacity of 427.5 mA h g^−1^ was still obtained, which was higher than the theoretical capacity of commercial graphite (372 mA h g^−1^). In addition, upon altering the current density back to 100 mA g^−1^ after high rate discharge–charge cycling, a capacity of 898.3 mA h g^−1^ could be recovered, indicating the great rate performance of NiO/Ni_3_V_2_O_8_ NPAs. In contrast, the capacities of NiO samples were much lower than these of NiO/Ni_3_V_2_O_8_ NPAs under the same tested conditions. The superior rate performance of the NiO/Ni_3_V_2_O_8_ NPAs could be closely related to their superior conductivity. In addition, NiO/Ni_3_V_2_O_8_ NPAs performance was exceptional for long cycling at a high current density of 1000 mA g^−1^. Fig. 5e showed that the reversible discharge capacity reached 570.1 mA h g^−1^ after 600 cycles. The CE of the NiO/Ni_3_V_2_O_8_ NPAs was as high as 99.9% within 600 cycles, demonstrating highly reversible Li-storage processes during long-term cycling.

**Fig. 6:**
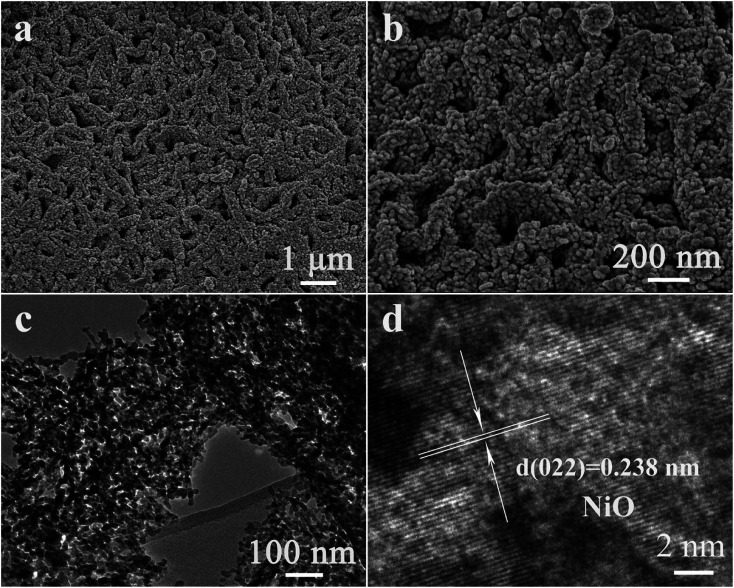
(a) The SEM, (b) TEM, (c) SEAD and (d) HRTEM images of NiO/Ni_3_V_2_O_8_ NPAs after 100 cycles with different magnification.

It appeared that the unique morphology and structure of the NiO/Ni_3_V_2_O_8_ NPAs after 100 cycles with charge were the key feature for the excellent electrochemical performance of the NiO/Ni_3_V_2_O_8_ NPAs. It was discovered that the morphology of cycled NiO/Ni_3_V_2_O_8_ NPAs was much different from the original morphology (Fig. 6a). The NiO/Ni_3_V_2_O_8_ NPAs after 100 cycles had a new film-like porous structure which assembled with numbers of nanoparticles as seen from a high magnification SEM image of NiO/Ni_3_V_2_O_8_ NPAs (Fig. 6b). TEM results (Fig. 6c) showed that the mean size of the most nanoparticle was around 10–30 nm, whereas the HRTEM image of cycled NiO/Ni_3_V_2_O_8_ NPAs showed that the crystal lattice spacing was 0.238 nm corresponding to the (022) plane for NiO, which was consistent with the results of *ex situ* X-ray (Fig. 4c-f). Such microstructural evolution along with the cycling process could be ascribed to a very unique and novel reconstruction process.^49^ It indicated that the initial size reduction of NiO/Ni_3_V_2_O_8_ NPAs produced numbers of nanoparticles, and the reassembly of these nanoparticles formed a secondary structure during cycling process. The newly formed film-like porous architecture might facilitate improve the reaction kinetics of the NiO/Ni_3_V_2_O_8_ NPAs, which was responsible for the outstanding lithium storage properties.

**Fig. 7:**
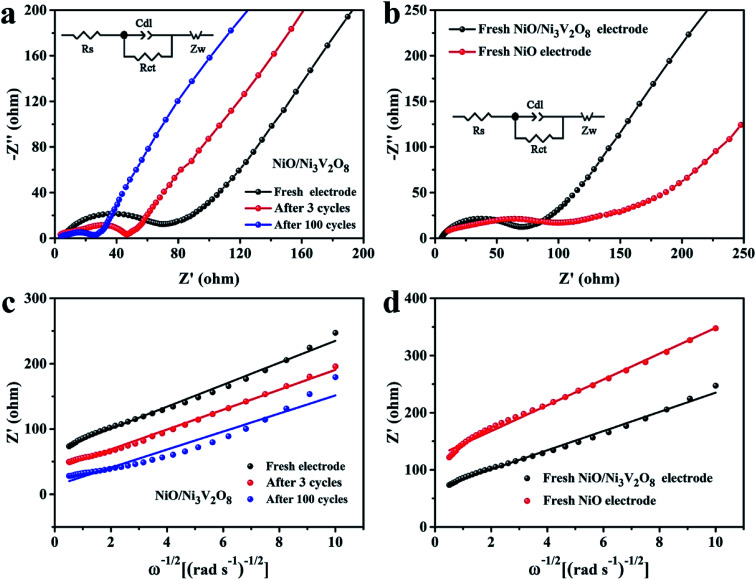
EIS spectra of (a) the NiO/Ni_3_V_2_O_8_ NPAs after different cycles and (b) fresh NiO/Ni_3_V_2_O_8_ NPAs and bare NiO samples (the insert was the equivalent circuit, where *R*_s_ is the SEI film and/or contact resistance, *R*_ct_ is the charge-transfer impedance on samples/electrolyte interface, *C*_dl_ is the capacitance related to the double layer, and *Z*_w_ represents the lithium-diffusion process within samples). The relationship between the real parts *Z*′ and *ω*^−1/2^ for (c) the NiO/Ni_3_V_2_O_8_ NPAs after different cycles, (d) fresh NiO/Ni_3_V_2_O_8_ NPAs and NiO samples.

The EIS (100 kHz to 0.01 Hz, 5 mV s^−1^) was employed to explore the origin of excellent electrochemical properties of NiO/Ni_3_V_2_O_8_ NPAs with various states (Fig. 7). The fresh and cycled NiO/Ni_3_V_2_O_8_ samples were fitted by equivalent circuit and the fitted results were summarized in Table S3.† Results revealed that *R*_s_ of the NiO/Ni_3_V_2_O_8_ samples increased slightly as the formation of SEI films produced by the morphology change of NiO/Ni_3_V_2_O_8_ NPAs in cycling. Meanwhile, *R*_ct_ decreased with the increase of cycle number, indicating the decrease of charge-transfer impedance, which might be associated with the novel electrochemical reconstruction as demonstrated in Fig. 6. The newly formed film-like porous architecture during the cycling process consisted of many small-sized NiO/Ni_3_V_2_O_8_ nanoparticles, which facilitated the electric contact between NiO/Ni_3_V_2_O_8_ NPAs and Ti foil, resulting in the enhanced electrochemical performance of the integrated NiO/Ni_3_V_2_O_8_ NPAs. Fig. 6b showed that the fresh NiO/Ni_3_V_2_O_8_ NPAs exhibited a lower *R*_s_ and *R*_ct_ than those of NiO samples. The reduction of resistance might derive from the presence of the Ni_3_V_2_O_8_ crystals. Fig. 7(c and d) displayed the relationship between the real parts *Z*′ of the impedance and *ω*^−1/2^ in the low frequency region, the Warburg factor *σ* could be calculated from the slope of the line *Z*′–*ω*^−1/2^ (*σ* = *Z*'/*ω*^−1/2^), which was inversely proportional to the diffusion coefficient of Li-ion.^52,53^ That was to say, the smaller the value of *σ* was, the higher the diffusion coefficient of Li-ions showed. It was seen that the Warburg factor *σ* for the NiO/Ni_3_V_2_O_8_ NPAs decreased slightly in value along with the cycling number (Fig. 7c, Table S3†), indicating enhanced Li-ion diffusion process. Both the reduced size and the favorable microstructural evolution in cycling might be responsible for the improved kinetics properties of the NiO/Ni_3_V_2_O_8_ NPAs. Furthermore, the Warburg factor *σ* was 16.82 for the fresh NiO/Ni_3_V_2_O_8_ NPAs and 22.59 for the fresh NiO electrode (Fig. 7d), suggesting that the kinetics property of NiO/Ni_3_V_2_O_8_ NPAs was better than that of fresh NiO electrode, which was consistent with the charge/discharge results.

As is discussed above, the outstanding electrochemical properties of NiO/Ni_3_V_2_O_8_ NPAs could be ascribed to several aspects. Firstly, the direct growth of hierarchical NiO/Ni_3_V_2_O_8_ nanostructures on Ti foil could ensure robust mechanical adhesion and short transportation length for both Li^+^ and electrons. Secondly, the NiO/Ni_3_V_2_O_8_ NPAs constructed an open and highly porous framework structure, which facilitate the electrolyte penetration and Li^+^ migration as well as the accommodation of strains during the cycling process. Thirdly, the novel electrochemical reconstruction of the NiO/Ni_3_V_2_O_8_ NPAs during the cycling process could favor to improve kinetics properties of conversion and intercalation reaction, leading to enhanced electrochemical performances. Moreover, the synergistic effect between NiO and Ni_3_V_2_O_8_ is not obviously neglectable, which can account for the superior performance of NiO/Ni_3_V_2_O_8_ NPAs.

## 4 Conclusions

We had successfully prepared a hierarchical NiO/Ni_3_V_2_O_8_ NPAs using a facile one-step hydrothermal method, followed by subsequent annealing treatment. The resultant NiO/Ni_3_V_2_O_8_ NPAs took an open and highly porous framework structure, assembled by interpenetrated nanoplatelets with the thickness of about 60–80 nm, in which the NiO and Ni_3_V_2_O_8_ subunits were homogeneously dispersed. Compared with the bare NiO electrode, the NiO/Ni_3_V_2_O_8_ NPAs rendered high reversible capacity of 1169.3 mA h g^−1^ after 100 cycles at 200 mA g^−1^, delivered 570.1 mA h g^−1^ after 600 cycles at 1000 mA g^−1^, and retained 427.5 mA h g^−1^ even at 8000 mA g^−1^. The superior lithium storage performance could be ascribed to the rationally designed composition and nanostructure of the NiO/Ni_3_V_2_O_8_ NPAs. The possible mechanism of electrochemical reaction of NiO/Ni_3_V_2_O_8_ NPAs would be the common conversion and intercalation reactions of nickel/vanadium based multiple oxides. These results suggested that NiO/Ni_3_V_2_O_8_ NPAs can be used as advanced electrodes for next generation LIBs. This work may open a new avenue to design and synthesis hierarchical nanostructure with a synergistic effect.

## Conflicts of interest

There are no conflicts to declare.

## Supplementary Material

RA-009-C9RA08252B-s001
