# Supplementary material for: Design and synthesis of hierarchical NiO/Ni3V2O8 nanoplatelet arrays with enhanced lithium storage properties
Source: RSC Adv. 2019 Dec 2;9(67):39536–44. doi: 10.1039/c9ra08252b (PMC9076189; doi:10.1039/c9ra08252b)
Supplement: RA-009-C9RA08252B-s001 [file RA-009-C9RA08252B-s001.pdf]

Electronic Supplementary Material (ESI) for RSC Advances.
This journal is © The Royal Society of Chemistry 2019

Electronic Supplementary Information

Design and synthesis of hierarchical $NiO/Ni_3V_2O_8$ nanoplatelet arrays with enhanced lithium storage properties

Yang Li*[a], Feng Duan[a], Shuai Yang[a], Qihuang Deng[a], Songli Liu[a] and Cheng Peng[a]

[a]*College of Materials Science and Engineering, Yangtze Normal University, Chongqing, 408100, People's Republic of China*

** Corresponding author. Tel.: +86-023-72790029, Fax: +86-023-72790029*
E-mail: yangli_yznu@163.com;

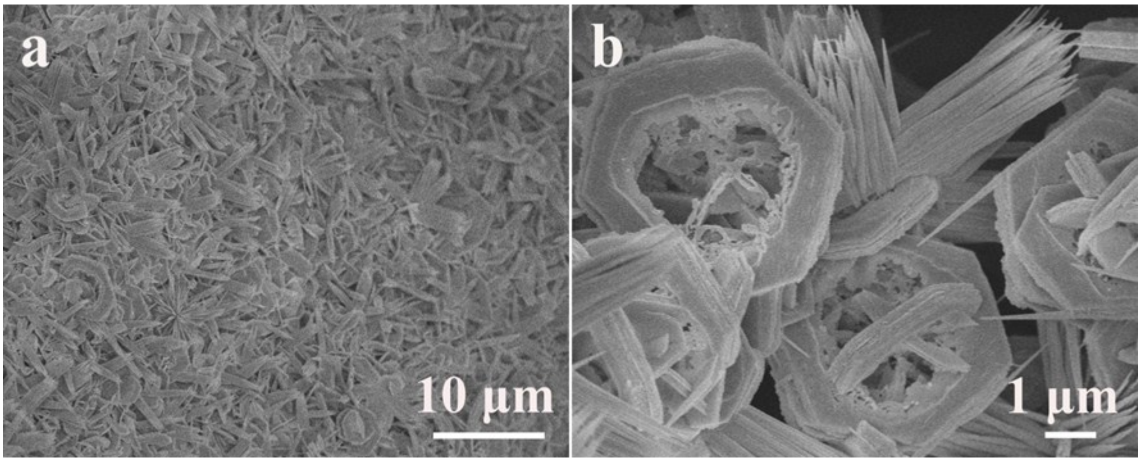

Fig. S1 SEM pattern of NiO samples with low magnification.

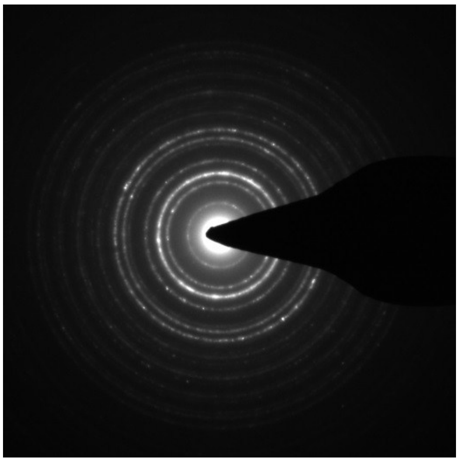

Fig. S2 The SAED patterns of $NiO/Ni_3V_2O_8$ NPAs

Table S1 Quantitative analysis of Ni and V contents of the samples by ICP-MS.

Samples	Ni %	V %	Ni/V mole ratio
$NiO/Ni_3V_2O_8$ nanocomposites	89.72	10.28	7.58:1

Table S2 The comparison of the capacity retention of $NiO/Ni_3V_2O_8$ NPAs with the reported results.

Nanomaterials	Current density (mA g^{-1})	Cycle Number	Capacity (mAh g^{-1})	Reference

$NiO/Ni_3V_2O_8$ NPAs	100	100	1169.3	This work
NiO-Co_3O_4 nanoplate composite	100	70	633	[41]
porous NiO-ZnO hybrid nanofibers	200	120	949	[42]
Hierarchical Fe_2O_3@NiO core/shell nanorod array	200	50	1047.2	[50]
Multilayer CuO@NiO Hollow Spheres	100	200	1061	[51]
Hierarchical mesoporous hybrid NiO-$MnCo_2O_4$ microspheres	200	50	846	[37]
$Ni_3V_2O_8$ amorphous wire encapsulated in crystalline tube nanostructure	300	300	962	[32]

Table S3 Impedance parameters calculated from equivalent circuit model.

	NiO (Fresh samples)	$NiO/Ni_3V_2O_8$ (Fresh samples)	$NiO/Ni_3V_2O_8$ (3rd)	$NiO/Ni_3V_2O_8$ (100th)
Rs (Ω)	6.5	5.1	8.1	13.8
Rct (Ω)	134.5	61.5	42.7	11.6
σ	22.59	16.82	15.24	13.82
